# Physiological Responses at 15 Minutes of Recovery after a Session of Functional Fitness Training in Well-Trained Athletes

**DOI:** 10.3390/ijerph19148864

**Published:** 2022-07-21

**Authors:** José Luis Maté-Muñoz, Mihai Budurin, Salvador González-Lozano, Juan Ramón Heredia-Elvar, Ana María Cañuelo-Márquez, Manuel Barba-Ruiz, Diego Muriarte, Manuel Vicente Garnacho-Castaño, Juan Hernández-Lougedo, Pablo García-Fernández

**Affiliations:** 1 Department of Radiology, Rehabilitation and Physiotherapy, Complutense University of Madrid, 28040 Madrid, Spain; jmate03@ucm.es (J.L.M.-M.); pablga25@ucm.es (P.G.-F.); 2 Department of Physiotherapy, Camilo José Cela University, Castillo de Alarcón 49, Villafranca del Castillo, 28692 Madrid, Spain; mihifisio@hotmail.com (M.B.); salva.p28@gmail.com (S.G.-L.); 3 Department of Physical Activity and Sports Science, Alfonso X El Sabio University, 28691 Madrid, Spain; jelvaher@uax.es (J.R.H.-E.); acanumar@uax.es (A.M.C.-M.); mruizbar@uax.es (M.B.-R.); 4 INEF-Department of Sports, Faculty of Physical Activity and Sport Sciences, Polytechnic University of Madrid, C/Martín Fierro 7, 28040 Madrid, Spain; diego.muriarte@upm.es; 5 Nursing Department, Campus Docent Sant Joan de Déu, University of Barcelona, 08034 Barcelona, Spain; manuelvicente.garnacho@sjd.edu.es; 6 IdISSC—Instituto de Investigación Sanitaria del Hospital Clínico San Carlos, 28040 Madrid, Spain

**Keywords:** sport performance, high intensity functional training, fatigue, strength, velocity, load, Crossfit, training, human performance

## Abstract

Background: the aim of this study was to analyse muscle fatigue and metabolic stress at 15 min of recovery after performing two independent sessions of functional fitness training (FFT): a session of strength functional fitness training (FFTstrength) and a session of endurance functional fitness training (FFTendurance). Methods: eighteen well-trained men conducted two protocols, separated by one week of rest: FFTstrength (3 sets of 21, 15 and 9 repetitions of Thruster with bar + Pull ups) and FFTendurance (3 sets × (30 kcal rowing + 15 kcal assault air bike)). Neuromuscular fatigue and metabolic stress were measured right before, right after and at 10 and 15 min after completing the FFT workout, as well as the mean heart rate (HRmean) and the rating of perceived exertion (RPE) at the end of the FFT. Results: FFTendurance recovered the velocity loss values after 15 min of recovery. On the other hand, FFTstrength only recovered velocity in the 1 m·s^−1^ Tests in squat (SQ), since the velocity levels were 7% lower in the 1 m·s^−1^ Tests in military press exercise (MP) after 15 min. Conclusions: These data indicate that there are specific recovery patterns not only as a function of the exercise and the body regions involved, but also regarding the recovery of neuromuscular and metabolic factors, since both FFT workouts obtained high blood lactate concentrations.

## 1. Introduction

High-intensity functional training, also known as Functional Fitness training (FFT), is a training modality based on different exercises of muscular strengthening executed in different movement planes (FFTstrength), such as Olympic and power lifting, calisthenic, plyometric and gymnastic exercises, and on intervallic exercises of aerobic training (FFTendurance), such as running, rowing, cycling, rope jumping, etc. [1,2]. FFT workouts are performed in circuits at high intensity, with the aim of completing a certain predetermined number of repetitions in the shortest time possible (“rounds for time” or “RFT”) or completing as many repetitions of a set of exercises as possible within a certain pre-set time (“as many rounds as possible” or “AMRAP”) [3,4]. Crossfit^®^ is a type of FFT [5,6].

The physiological responses of this training modality have been analysed in different studies, showing high values of heart rate (HR) (90–95% HRmax), rating of perceived exertion (RPE) (RPE values > 8/10, RPE values > 15/20) and blood lactate concentration (>10 mmol·L^−1^) [2,7,8,9]. Moreover, acute cardiovascular responses do not vary among “workouts of the day” (WOD) based on the two most used training structures: RFT vs. AMRAP (10). However, significantly higher blood lactate levels have been reported using the RFT methodology right at the end and 30 min after the exercise, indicating different metabolic demands [10]. Due to this high intensity, high levels of muscle fatigue have also been detected [loss of jump height in countermovement jump (CMJ) > 6.5%] right after completing Crossfit^®^ WOD [8,9]. Some case studies have documented that certain people who carried out Crossfit^®^ for several consecutive days had health complications due to muscle damage [11,12,13]. Other studies have also explored the recovery of biochemical parameters (hepatic transaminases, blood glucose and creatine phosphokinase) after performing a Crossfit^®^ session, showing that all of these parameters returned to baseline levels at 48 h after presenting a significant increase produced by the completion of the WODs [14]. In this line, Tibana et al. (2016) [15] analysed the effects of two consecutive Crossfit^®^ sessions (separated by 24 h) on cytokines interleukin 6 (IL-6) and interleukin 10 (IL-10) and muscle power, reporting a decrease of IL-10 after the two consecutive Crossfit sessions. This suggests that it may be necessary to incorporate sessions of lower intensity, or rest days between sessions [15].

The interest in knowing the recovery times after an exercise session, leads us to ask the following question: how long does it take to recover pre-exercise values after a FFT workout in the same day? In this regard, García-Fernández et al. (2021) [2] analysed muscle recovery up to 20 min after conducting a single FFT workout in trained adults. Their results showed a significant decrease in jumping capacity until minute 10 after FFT workout. However, after 20 min of recovery, the values began to recover without reaching the baseline levels [2]. However, they did not find the exact point where the values start to recover, which could be slightly above 10 min, at the midpoint (between 10 and 20 min) and slightly below 20 min. To be able to calculate that point where recovery begins more accurately, it would be possible to determine an approximate rest to face another FFT workout with a recovery that guarantees the lowest residual fatigue and deterioration of the performance capacity, since muscle fatigue could reduce the exercise execution velocity, thereby altering the correct biomechanics and increasing the risk of injury [16]. Nevertheless, although the quantification of muscle fatigue in the study of García-Fernández et al. (2021) [2] was measured through the widely used method of countermovement jump (CMJ), this method only assesses the mechanical variables related to the lower limbs, and it does not gather information about muscle fatigue in the upper limbs. Since the entire body is used in the FFT workouts, it is important to be able to measure muscle fatigue in the whole body, using, for example, the changes of velocity with respect to 1 m·s^−1^ of the mean propulsive velocity (MPV) [17].

Therefore, the aim of this study was to analyse muscle fatigue and metabolic stress at 15 min of recovery after performing two independent sessions of FFT: one session of strength training (FFTstrength) and one session of endurance training (FFTendurance).

## 2. Materials and Methods

### 2.1. Study Design

**Figure 1:**
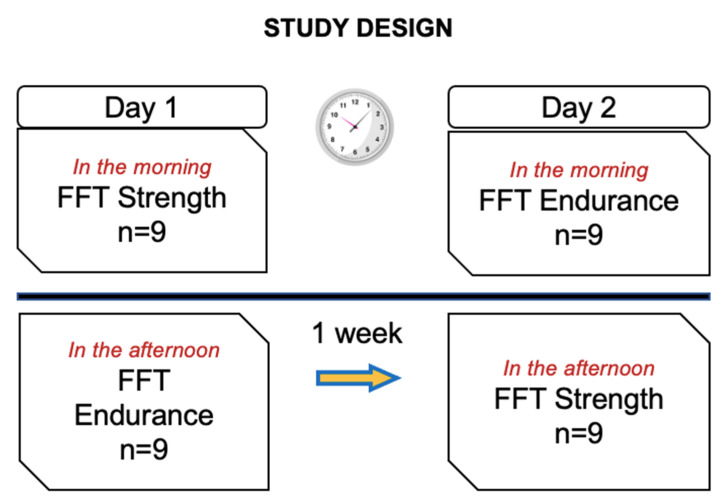
Study design.

This was a crossover study in which two different FFT protocols were performed, separated by one week. One session was carried out with strength exercises (FFTstrength) and the other with endurance exercises (FFTendurance). Neuromuscular fatigue and metabolic stress were measured before and at three time points after the FFT, as well as mean and final heart rate and rate of perceived exertion (RPE) at the end of the FFTs. All tests were conducted in the cross-training gym, where Ruster^®^ calibrated material is available. In session 1 (day 1), half of the participants completed either the FFTstrength or the FFTendurance. The following week, subjects did the session they did not complete on the first day (day 2). No participants in the FFTstrength coincided at the gym with those completing the FFTendurance and vice-versa (Figure 1). The FFTstrength and the FFTendurance were performed one week later, on the same day of the week. Participants performed the FFT workouts within the same timeframe (±2 h) (morning or afternoon), in order to control for the effects of the circadian rhythm [18], and under the same conditions of temperature (24–26 °C), atmospheric pressure (1011–1020 hPa) and humidity (60–80%).

### 2.2. Participants

Eighteen healthy strength-trained men (with age, height, weight, body mass index (BMI) and body fat (%) of 24.22 ± 2.73 years, 76.43 ± 8.22 kg, 176.06 ± 4.49 cm, 24.55 ± 2.21 kg/m^2^, and 13.62% ± 3.27, respectively) participated in the study. A body composition analyser was used to determine body weight and estimate body fat percentage (Tanita BC-601, Tanita Corp., Tokyo, Japan). The sample size calculation was done with α = 0.05 (5% chance of type I error) and 1 − β = 0.75 (power 75%), applying the results of previous studies, in which the sample size was the equal or smaller. The calculated sample size was 16 strength-trained subjects. The subjects were instructed not to take psychotropic substances and/or narcotic, performance-enhancing drugs, nutrient supplements or stimulants. Furthermore, exclusion criteria were cardiovascular, metabolic, lung and neurological diseases or any orthopaedic limitation that may restrict their performance or the correct execution of the training exercises. No elite athletes participated.

Subjects signed the informed consent form after being informed about the study design. The study protocol was approved by the University’s ethics committee according to the tenets of the Helsinki declaration [19]. The participants were encouraged to perform their usual training the week before both FFT protocols, except the day before each FFT protocol, when they were requested to rest. In addition, they were instructed not to consume any food or drink containing alcohol, caffeine or other stimulants within 2 h prior to the execution of the FFT protocols.

### 2.3. Functional Fitness Training

**Figure 2:**
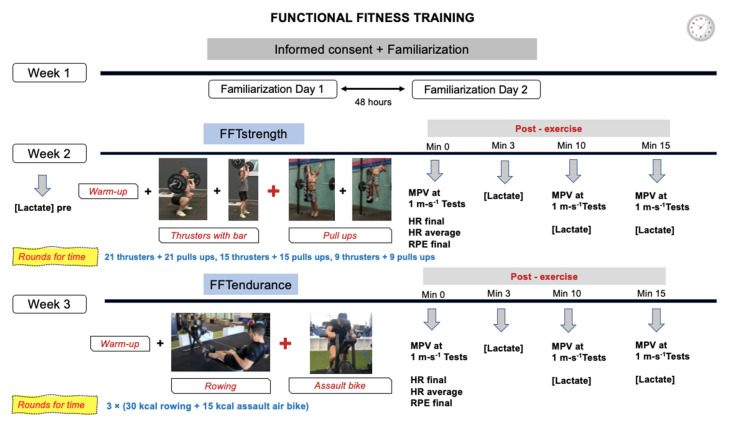
FFT = functional fitness training; MPV = Mean propulsive velocity; Min = minutes; HR = Heart rate; RPE = Rate of perceived exertion.

Two strength exercises were used in the FFT strength session (FFTstrength): (1) thruster with bar, and (2) pull ups using the butterfly technique. In the thruster exercise, the subject moves an object in one fluid motion from ground to shoulders while squatting below parallel. The thruster is a combined movement that consists of a front squat (the subject, holding weight in front of his/her shoulders, squats down below parallel and returns to standing) and a push press (the subject moves the weight from his/her shoulders over his/her head; the subject is allowed to dip and drive the object with his/her legs). The thruster starts with the bar on the shoulders. The bar shall be brought to the shoulders with a power clean. In order to correctly perform the pull ups, it was mandatory to put the chin over the bar. In this study, the bar weighed 35 kg. One of the evaluators checked that the execution of this exercise was correct, shouting “not correct” when the execution was wrong, in which case the repetition was repeated. Three sets of each exercise were alternated, performing 21 repetitions in set 1, 15 repetitions in set 2 and 9 repetitions in set 3. The complete sequence was: (21 thrusters + 21 pull ups, 15 thrusters + 15 pull ups, 9 thrusters + 9 pull ups) (Figure 2).

For the FFT endurance session (FFTendurance), two exercises were performed: (1) rowing and (2) cross training bike (assault bike). The exercises were alternated, performing 3 sets to complete 30 kilocalories (kcal) in rowing and 3 sets of 15 kcal in assault air bike. The complete sequence was: 3 × (30 kcal rowing + 15 kcal assault air bike). The rowing machine used was Concept 2^®^ D PM5 (Concept2, Inc., Morrisville, VT, USA) and the cross-training bike used was Assault AirBike (Assault Fitness, Carlsbad, CA, USA).

The “rounds for time” (RFT) methodology was used for both workouts and consisted of performing the same sequence of exercises in an all-out manner in the shortest time possible without restrictions. The exercises for the workouts were performed on the movement standards defined by the International Functional Fitness Federation (iF3) published in January 2022. (https://functionalfitness.sport/sport/movement-standards/) (accessed on 31 May 2022).

A familiarisation period of exercises was performed the week before the tests, separated by 2 days (Figure 2). In addition, prior to each FFT protocol, the participants performed a warm-up of 5 min of cardiovascular work, 5 min of joint mobility, ballistic stretching and strength exercises.

### 2.4. Mechanical Measurements of Fatigue

To evaluate the fatigue generated by the FFTs protocols, the percent change between pre-post exercises was used with an individual load that could be lifted at ~1 m·s^−1^ of the MVP (MPV at 1 m·s^−1^ Tests) in a military press exercise (MP) and in a squat (SQ). To obtain the individual load at 1 m·s^−1^, weights were lifted until this velocity was reached. The subjects initiated with the barbell (20 kg) and progressively increased the weight by 5 kg, completing 3 repetitions with each load, resting 3 min between loads. Sánchez Medina and González-Badillo (2011) [17] chose the value of 1 m·s^−1^ because it is a sufficiently high velocity, which is attained against medium loads (45–50% 1RM in MP and ~60% 1RM in SQ), and it allows a robust expression of the effect of loading on velocity, as well as being a relatively easy-to-move and well-tolerated load. The mean MPV of the three repetitions before exercise was contrasted with the mean MPV of the three post exercise repetitions [17]. All repetitions were carried out at maximum velocity. The MPV at 1 m·s^−1^ Squat and Military Press Tests was assessed (bar velocity values during the propulsive phase, defined as the portion of the concentric phase during which bar acceleration is ≥9.81 m·s^−2^). Loss of MPV at 1 m·s^−1^ Squat and Military Press Tests were calculated as percentage (%) between pre-exercise and post-exercise (min 0, min 10, min 15).

A Smith machine (Multipower) (Matrix, Chácara Alvorada, Brazil) was used in the MPV at 1 m·s^−1^ Tests. In this set up, both ends of the barbell are fixed, allowing only for the vertical movement of the bar. In order to estimate the execution velocity of each repetition in different tests, a previously validated isoinertial dynamometer was used (Speed4LifTM, Madrid, Spain) [20]. This isoinertial dynamometer consists of a cable-extension linear position transducer attached to the bar. Data were directly recorded by the differentiation of the displacement data with respect to time at a sampling rate of 1000 Hz via Wi-Fi connection to an Android smartphone using the Speed4Lift application v.4.1. The cable was vertically attached to one side of the bar by means of a Velcro strip.

### 2.5. Blood Lactate Concentrations

Before and after the FFT protocol (Min 3 post-exercise/Min 10 post-exercise/Min 15 post-exercise), blood lactate samples (5 μL) were obtained from the fingertip. A pre-calibrated and validated portable analyser, Lactate Pro 2 LT-1710 (Arkray Factory Inc., KDK Corporation, Shiga, Japan), was used to determine blood lactate concentrations [21,22].

### 2.6. Ratings of Perceived Exertion

RPE was obtained just after finishing the FFT protocols by asking participants to describe the hardness of their overall training [23], by grading their level of exertion on a Borg scale CB-10 from 0 to 10—from “very light” to “maximum exertion”. Each subject indicated with their finger, on a scale of size DIN-A3, how hard the FFT workout was for them. The subjects were trained to avoid verbal descriptions and to just point directly at the scale with their finger.

### 2.7. Heart Rate

Mean HR during FFT protocols and HR right after completing the exercise were calculated by telemetry (RS-800CX, Polar Electro OY; Kempele, Finland).

### 2.8. Statistical Analysis

The Shapiro–Wilk test was used to check the normality of the data. A two-way ANOVA with repeated measures—protocols × measures (FFTstrength and FFTendurance × pre-exercise/Min 0 post-exercise/Min 10 post-exercise/Min 15 post-exercise)—was performed to compare the effects of the two experimental conditions (FFTstrength and FFTendurance). Greenhouse–Geisser probability levels were used to check for sphericity, and Bonferroni adjustments were employed to control for multiple post-hoc comparisons. Linear regressions were applied to establish the relationships between loss of velocity values and blood lactate. To detect differences in heart rate and RPE between FFTs, Student’s *t* test for related measures was applied. All data were expressed as means and standard deviations (SD), with 95% confidence intervals (CI). In addition, we determined the effect size, known as partial eta-squared (ηp^2^), which was classified into trivial (ηp^2^ ≤ 0.01), small (0.01 ≤ η_p_^2^ < 0.06), moderate (0.06 ≤ η_p_^2^ < 0.14) or large (η_p_^2^ ≥ 0.14) [24], along with the statistical power (SP). The percentage of loss of velocity was calculated using the following equation: post−pre/pre × 100. The level of significance was set at *p* < 0.05. All statistical tests were performed using the package SPSS version 25.0 (SPSS, Chicago, IL, USA).

## 3. Results

Table 1 shows the descriptive data for FFT times and MVP at 1 m·s^−1^ Squat and Military Press test.

**Figure 3:**
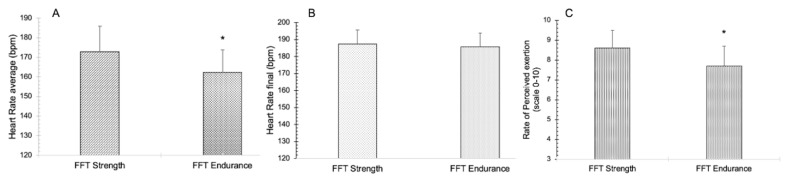
Comparison between FFTs for the variables Heart Rate average (**A**), Heart Rate final (**B**), and Rate of Perceived Exertion (**C**); bpm = beats per minute; * = significant difference (*p* < 0.05).

For the mean heart rate and subjective perception of effort, significant differences were detected between groups (*t* = 2.554, *p* = 0.015, *t* = 2.752, *p* = 0.009, respectively), whereas, for final heart rate, no significant differences were found (*t* = 0.571, *p* = 0.572) (Figure 3).

For MVP at 1 m·s^−1^ Squat Test, MVP at 1 m·s^−1^ Military Press Test and blood lactate, significant differences were identified in time (*p* < 0.01) between the FFT protocols (*p* < 0.01) and the time × FFT protocols interaction (*p* < 0.01) (Table 2). For MVP at 1 m·s^−1^ Squat Test, in the pairwise comparison, a significantly lower velocity was observed in Min 0 post-exercise only for FFTstrength (*p* = 0.002), and in Min 0 post-exercise with respect to Min 10 post-exercise and Min 15 min post-exercise in both FFTs (FFTstrength, *p* = 0.046 and *p* = 0.015; FFTendurance, *p* = 0.004 and *p* = 0.039, respectively). However, for MVP at 1 m·s^−1^ Military Press Test, FFTstrength showed significant differences in the pre-exercise and in Min 0 post-exercise with the rest of the time points (*p* < 0.01), whereas FFTendurance presented statistical significance in the pre-exercise with Min 0 post-exercise (*p* = 0.011) and in Min 15 post-exercise with Min 0 post-exercise (*p* = 0.013) and Min 10 post-exercise (*p* = 0.023). For the blood lactate concentrations, differences were observed among all pre- and post-exercise measurements (*p* < 0.01), whereas, for the FFT protocols, significant differences were found in the three post-exercise measurements (*p* < 0.01) (Table 2)

The percentage of loss of velocity in MVP at 1 m·s^−1^ Test is shown in Table 3. As can be observed in the squat test, for FFTstrength, the loss of velocity in the post-exercise at 0 min was 8.9%, whereas, in the post-exercise at 15 min, the loss of velocity was almost 4%. Nevertheless, this variable, for FFTendurance, did not exceed 4% at the end of the exercise (post 0 min), recovering the pre-exercise values after 15 min of rest. In the Military Press Test, the loss of velocity was greater in FFTstrength right after completing the exercise (−18%), remaining at −7% after resting for 15 min. However, in FFTendurance, there was only a loss of 6% at the end of the exercise, practically recovering the values after 15 min of rest (−2%). The values of blood lactate were similar in both FFT protocols, maintaining a high metabolic stress after resting for 15 min (FFTstrength = 13.76 mmol·L^−1^; FFTendurance = 12.55 mmol·L^−1^).

**Figure 4:**
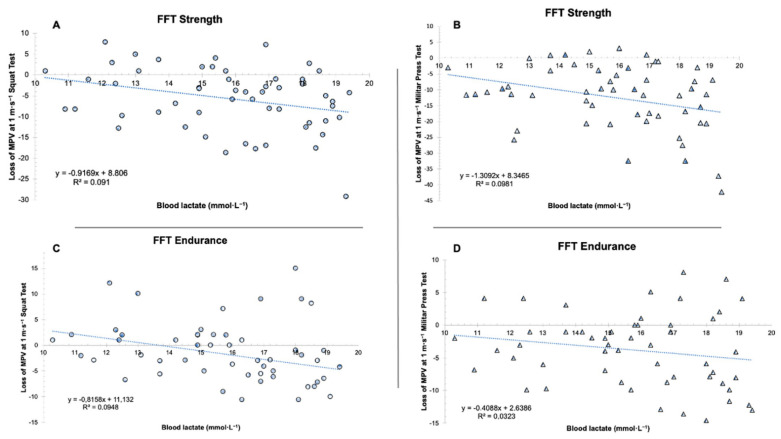
(**A**) Relationship between loss of MPV at 1 m·s^−1^ Squat Test and blood lactate in FFTstrength (*R*^2^ = 0.091, *p* = 0.027), (**B**) relationship between loss of MPV at 1 m·s^−1^ Military Press Test and blood lactate in FFTstrength (*R*^2^ = 0.0981, *p* = 0.021), (**C**) relationship between loss of MPV at 1 m·s^−1^ Squat Test and blood lactate in FFTendurance (*R*^2^ = 0.0948, *p* = 0.024), (**D**) relationship between loss of MPV at 1 m·s^−1^ Military Press Test and blood lactate in FFTendurance (*R*^2^ = 0.0323, *p* = 0.194).

There was no correlation between the loss of velocity in MPV at 1 m·s^−1^ and blood lactate concentrations in the Squat and Military Press tests. In both FFTs, the values of MPV were recovered, whereas those of blood lactate remained very high (Figure 4).

## 4. Discussion

After performing two FFT workout protocols (FFTstrength and FFTendurance) and recovering for 15 min, the main finding of this study was that, while MPV was recovered at 1 m·s^−1^ Tests in the SQ test in both FFTstrength and FFTendurance, the values of MPV at 1 m·s^−1^ Tests in the MP test were not recovered in FFTstrength. Moreover, a very high metabolic stress was detected at 15 min of recovery in both FFT workouts (>12 mmol·L^−1^ at 15 min post-exercise), with significantly higher lactate levels in FFTstrength (17.85 ± 1.18 mmol·L^−1^ VS. 16.68 ± 1.63 mmol·L^−1^). Therefore, this recovery of execution velocity after 15 min of rest is not correlated with the high metabolic stress.

In the analysis of the execution time of the FFTworkouts, it was observed that the mean duration of FFTendurance was almost twice as long as that of FFTstrength (496 s VS. 291 s). Nevertheless, the mean heart rate (HR_mean_) and RPE were significantly higher in FFTstrength (FFTstrength, HR_mean_ = 172.8 lpm ± 13.1, RPE = 8.6 ± 0.9; FFTendurance; HR_mean_ = 162.3 ± 11.4 lpm, RPE = 7.7 ± 1). Comparing the values of HR and RPE obtained with FFTstrength in other studies, we can assert that they were similar to those found in another strength WOD (5 min power clean with a load of 40% maximum power obtained in the one repetition maximal test) [9]. The study of Fernández-Fernández et al. (2015) [7], which was conducted with the same FFTstrength as in the present study, obtained similar values of HR_mean_ (179 ± 8.4 lpm), and RPE (8.4 ± 0.9). However, the blood lactate concentrations were higher in our study (17.85 ± 1.18 mmol·L^−1^ VS. 14.0 ± 3.3 mmol·L^−1^), with an even greater difference considering that the initial values were different (1.68 ± 0.31 mmol·L^−1^ VS. 4.0 ± 1.3 mmol·L^−1^) [7]. In another study, which compared recovery in 48 h for two modalities of WODs (AMRAP VS. RFT), the values of HR_final_, RPE and blood lactate obtained with RFT were more similar to those found in the present study (HR_final_ = 184.2 ± 8.6 lpm; RPE = 8.2 ± 0.4; blood lactate = 18.38 ± 2.02 mmol·L^−1^) [14].

Furthermore, comparing the values obtained in FFTendurance with those of other studies, it was observed that the cardiovascular response of FFTendurance at the end of the exercise was lower than that of other metabolic conditioning WODs (8 set × 20 s with 10 s rest between sets, skip rope double unders) (HR_mean_ = 162.3 ± 11.4 lpm; VS. 178 ± 9 lpm, respectively) [9]. This could be due to a greater recovery interval between the change from rowing to cross training cycling in FFTendurance. Comparing such HR_mean_ with different FFT workouts, such as “Cindy” (sequence of 5 pull-ups, 10 push-ups and 15 air squats for 20 min), lower values were also observed [7,9,25]. Nevertheless, in another study that compared a “Cindy” (AMRAP) with a RFT (Open 18.4) [10], HR_mean_ was approximately 150 lpm in both WODs, being lower than the HR_mean_ obtained in the present study (162.3 ± 11.4 lpm). Thus, considering that our sample was constituted by subjects with experience in this type of training, a possible explanation in view of such differences could be the number of rounds completed in the “Cindy”. Nevertheless, the blood lactate concentrations were very high 3 min after completing the exercise (16.68 ± 1.63 mmol·L^−1^) with respect to other metabolic conditioning WODs (10.37 ± 2.91 mmol·L^−1^) [9], “Cindy” WODs (14.5 ± 3.2 mmol·L^−1^) [7], (~10 mmol·L^−1^) [10] or FFT workout with or without prefixed rest (~13.5–15 mmol·L^−1^) [4].

Comparing the metabolic stress produced between both sessions (FFTstrength and FFTendurance) during the 15 min of recovery, significant differences were observed at minute 3, minute 10 and minute 15 post-exercise, with higher levels of blood lactate in FFTstrength (Post-exercise 15 min = 13.76 ± 2.05 mmol·L^−1^ VS. 12.55 ± 1.65 mmol·L^−1^). A study that measured blood lactate at 30 min in two Crossfit^®^ WODs reported values of ~4 mmol·L^−1^ in an AMRAP and ~7 mmol·L^−1^ in an RFT, finding differences between these two training methodologies [10]. With respect to the present study, this indicates the great glycolytic dependency of both exercises, and that this accumulation of lactate in blood could lead to a decrease of muscle contraction [26,27], due to the accumulation of hydrogen ions, which in turn decrease the pH and generate metabolic acidosis, being an indirect precursor of muscle fatigue [28].

However, comparing this high metabolic stress with the execution velocity in the performance of the Squat and Military Press tests with the load of 1 m·s^−1^ after 15 min of recovery, it was observed that, for FFTendurance, the pre-exercise values were recovered, whereas, for FFTstrength, these values were not recovered only in the 1 m·s^−1^ Military Press Tests (−7% loss velocity). This could be due to the exercises used in FFTstrength, since the muscle involvement of the upper limbs may be very high (push press and pull ups), requiring more recovery time [29,30]. The loss of MPV in the post-exercise right after completing the exercise was 18%, whereas, in the 1 m·s^−1^ Squat Test, the loss of velocity was half (9%) (only one leg exercise was involved), although this was not significant (*p* > 0.05).

Furthermore, the loss of velocity was not correlated with blood lactate concentrations, neither in the 1 m·s^−1^ Squat Test nor in the 1 m·s^−1^ Military Press Test, in any of the FFTs. That is, the lactate concentrations were very high for 15 min of recovery, whereas the execution velocity returned to pre-exercise values, except in FFTstrength in the 1 m·s^−1^ Military Press Test (−7% loss velocity). Thus, based on the results of this study, it could be asserted that, although the blood lactate concentrations were very high during recovery, a cause-effect relationship with muscle fatigue cannot be established. This assertion is in line with the conclusions of other authors [31]. Muscle fatigue has been traditionally defined as the loss of capacity to generate strength with the inability to maintain the expected exercise level [32,33], or as the decrease of muscle shortening rate, thereby increasing the relaxation time [34]. Therefore, when the subject moves a certain load at a velocity of 1 m·s^−1^ before the exercise, and then moves the same load at a considerably lower velocity after the exercise, it is considered that is in a situation of muscle fatigue, as he/she applies less strength for the same load [17]. In this case, except for the 1 m·s^−1^ Military Press Test of FFTstrength, all the velocity values after 15 min practically returned to the pre-exercise values. This suggests that the neuromuscular capacity to generate a maximum strength peak could recover more rapidly. Nevertheless, the capacity to maintain the strength levels for a certain period of time (e.g., a FFT workout) may require a longer time to recover. In addition, this could explain the differences found between mechanical factors (load of 1m·s^−1^) and metabolic factors (lactate), which are probably linked to the fact that the first factor may be rather conditioned by the fatigue generated by factors such as inorganic phosphate accumulation [35], alteration of the calcium levels [36] and restoration of the phosphagen deposits in relatively short times (5 min) [37], whereas the second factor does not seem to explain, by itself, their direct relationship with fatigue.

García-Fernández et al. (2021) [2] analysed the effect of recovery from a FFT workout for 20 min through CMJ. The results showed that there was a decrease in the jumping capacity at minute 4 and at minute 10 post-exercise. However, the values began to be recovered at minute 20 [2]. This involves speculating about the exact point where recovery is established. To this end, in our study, the recovery of the pre-exercise values in FFTendurance and in the Squat Test of FFTstrength was obtained at minute 15, which is the intermediate point between minute 10 and minute 20 of the mentioned study.

Although the data of this study indicate what occurred minutes after performing the FFT workouts, it is necessary to know the physiological reaction of the body if it were subjected to another FFT workout after 15 min of recovery. Would the number of repetitions be the same at the same exercise intensity in the second FFT workout as in the first session? Moreover, if a third FFT workout were conducted in the same training session, how long would the subject have to rest? Would the high metabolic stress affect the execution of a second FFT workout?

Thus, further research is necessary to clarify the responses of the human body to the recovery of this type of exercises, as well as the time required to be in optimal condition to perform another FFT workout. The use of execution velocity in this study as a mechanical variable to measure muscle fatigue is fundamental in this type of FFT workouts, since a decrease of movement velocity is produced by muscle fatigue, which could modify the biomechanics of the exercise, thereby altering the execution technique [16] and increasing the risk of injury, as the exercises performed in FFT have high technical demands [8].

There is an increasing number of subjects who carry out this type of training. In addition, there was a great difference in the levels of physical condition, strength, resistance to fatigue and capacity to recover of these participants; thus, it was important for them to adapt both the duration of the FFT workout and the intra- and inter-session recovery. Therefore, further studies should be conducted with different training levels. Furthermore, few studies have been conducted in women. It would be interesting to consider similar studies in a sample of women to check if there are differences in the results compared to men. Lastly, although the participants were well trained in this type of training sessions, the sample was probably scarce.

According to the data from this study, as a practical application for trainers who perform these types of FFT workouts, after a 15-min rest, efforts could be made where the application of force is optimal, due to the contractile recovery of the muscle. However, FFT workouts would have to be efforts of short duration, since the high metabolic stress will prevent these levels of strength from being maintained over time, due to the non-replenishment of the glycolytic pathway. Another possibility would be to perform FFT workouts where there is no high fatigue, including recovery times between sets or rounds, which would allow blood lactate values to decrease.

## 5. Conclusions

While FFTendurance obtained a slight loss of velocity right after completing the exercise, the initial values were practically recovered after 15 min of rest. However, FFTstrength only recovered velocity in the 1 m·s^−1^ Tests in SQ, since the velocity levels were 7% lower in the 1 m·s^−1^ Tests in MP after 15 min. In view of these results, there may be specific recovery patterns not only as a function of the exercise and the body regions involved, but also in terms of the recovery of neuromuscular factors, with respect to metabolic recovery, since both FFT workouts had high blood lactate concentrations, which were >12 mmol·L^−1^ after 15 min of recovery. This should be taken into account for the design of later FFTworkouts.

## Figures and Tables

**Table 1:** Descriptive analysis of FFT times and MVP at 1 m·s^−1^ Squat and Military Press Test of the 18 study participants.

Variable	(M ± SD)
Time FFTstrength (s)	291 ± 110
Time FFTendurance (s)	496 ± 62
MVP at 1 m·s^−1^ Squat Test Load (kg)	55.39 ± 10.51
MVP at 1 m·s^−1^ Military Press Test Load (kg)	34.22 ± 7.46

FFT = functional fitness training; s = seconds, M ± SD = mean ± standard deviation.

**Table 2:** Analysis of the variable velocity and blood lactate before and after performing the strength and endurance FFT protocols.

**Variable**	**FFT Group**	**Pre-Exercise** **(M ± SD, 95% CI)**	**Post-Exercise 0 min** **(M ± SD, 95% CI)**	**Post-Exercise 10 min** **(M ± SD, 95% CI)**	**Post-Exercise 15 min** **(M ± SD, 95% CI)**	***p* Time** **η_p_^2^** **SP**	***p* Group** **η_p_^2^** **SP**	***p* Time × Group** **η_p_^2^** **SP**
MVP at 1 m·s^−1^ Squat Test (m·s^−1^)	Strength	1.01 ± 0.04 & (0.99–1.03)	0.92 ± 0.09 £ † (0.88–0.96)	0.96 ± 0.08 £ (0.93–0.99)	0.97 ± 0.07 £ (0.95–1.00)	0.001 * 0.356 0.964	0.002 * 0.426 0.917	0.008 * 0.206 0.849
	Endurance	1.01 ± 0.03 (0.99–1.03)	0.97 ± 0.07 # (0.93–1.01)	1.00 ± 0.06 (0.97–1.03)	1.01 ± 0.04 (0.98–1.04)			
MVP at 1 m·s^−1^ Military Press Test (m·s^−1^)	Strength	1.00 ± 0.04 † (0.99–1.02)	0.82 ± 0.1 £ † (0.78–0.86)	0.89 ± 0.07 £ (0.87–0.92)	0.93 ± 0.08 £ (0.90–0.96)	<0.001 * 0.613 1.000	<0.001 * 0.721 1.000	<0.001 * 0.456 1.000
	Endurance	1.00 ± 0.02 & (0.99–1.02)	0.94 ± 0.06 (0.90–0.98)	0.96 ± 0.04 (0.94–0.99)	0.98 ± 0.03 $ (0.95–1.01)			
**Variable**	**FFT group**	**Pre-exercise** **(M ± SD, 95% CI)**	**Post-exercise 3 min** **(M ± SD, 95% CI)**	**Post-exercise 10 min** **(M ± SD, 95% CI)**	**Post-exercise 15 min** **(M ± SD, 95% CI)**	***p* Time** **η_p_^2^** **SP**	***p* Group** **η_p_^2^** **SP**	***p* Time × Group** **η_p_^2^** **SP**
Blood lactate (mmol·L^−1^)	Strength	1.68 ± 0.31 † (1.55–1.82)	17.85 ± 1.28 £ † (17.15–18.55)	15.82 ± 2.00 £ † (14.92–16.72)	13.76 ± 2.05 £ (12.86–14.65)	<0.001 * 0.982 1.000	<0.001 * 0.623 0.999	0.008 * 0.241 0.829
	Endurance	1.57 ± 0.24 † (1.44–1.71)	16.68 ± 1.63 † (15.98–17.38)	14.77 ± 1.74 † (13.87–15.67)	12.55 ± 1.65 (11.66–13.44)			

MVP = Mean propulsive velocity; FFT = Functional fitness training; min = minutes. M = mean ± SD = standard deviation; CI = confidence intervals; η_p_^2^ = partial eta-squared; SP = statistical power. * = significant difference (*p* < 0.05). £ = significant difference between FFTs (*p* < 0.05). † = significant difference between all exercise times (*p* < 0.05). & = significant difference between pre-exercise and post-exercise at 0 min. **#**= significant difference between post-exercise at 0 min, post-exercise at 10 min and post-exercise at 15 min (*p* < 0.05). $ = significant difference between post-exercise at 15 min, post-exercise at 0 min and post-exercise at 10 min (*p* < 0.05).

**Table 3:** Analysis of the variable velocity and blood lactate before and after performing the strength and endurance FFT protocols.

**Variable**	**FFT**	**Post 0 min–Pre**	**Post 10 min–Pre**	**Post 15 min–Pre**
% Loss of MVP at 1 m·s^−1^ Squat Test (%)	Strength	−8.91	−4.95	−3.96
	Endurance	−3.96	−1	0
% Loss of MVP at 1 m·s^−1^ Military Press Test (%)	Strength	−18	−11	−7
	Endurance	−6	−4	−2
		**Post 10 min–Post 3 min**	**Post 15 min–Post 3 min**	**Post 15 min–Post 10 min**
Blood lactate(%)	Strength	−11.37	−22.91	−13.02
	Endurance	−11.45	−24.76	−15.03

MVP = Mean propulsive velocity; FFT = Functional fitness training; min = minutes.
